# Characterising nosing behaviours in pigs after mixing using social network analysis

**DOI:** 10.1016/j.animal.2025.101585

**Published:** 2025-08

**Authors:** S.L. Jowett, M.J. Silk, V. Lee, S.P. Turner, I. Camerlink

**Affiliations:** a Institute of Genetics and Animal Biotechnology of the Polish Academy of Sciences, Postępu 36a, 05-552 Jastrzębiec, Poland; b Institute of Ecology and Evolution, School of Biological Sciences, University of Edinburgh, Edinburgh, UK; c Animal Behaviour & Welfare, Animal and Veterinary Sciences Department, Scotland’s Rural College (SRUC), West Mains Rd., Edinburgh EH9 3jG, UK

**Keywords:** Contact behaviour, Exponential Random Graph Models, Social behaviour, Social network analysis, Sus scrofa

## Abstract

Affiliation as indicated by proximity has, to date, been used as the principal measure of positive relationships in farm animals. However, intensive housing may present a caveat to using proximity as a representation of affiliation as animals may be forced into proximity by stocking densities. To investigate affiliation patterns, this study examined differences in the expression of proximity and contact behaviours following regrouping in pigs. Animals (61 males and 56 females) were observed across eight groups (14.6 ± 0.69 SD pigs/group). Each group comprised a mix of familiar and unfamiliar finishing pigs (12 weeks old). Video observations occurred for two consecutive days following regrouping. Individuals were continuously observed for social nosing behaviour for 2 h in total, balanced across days. Social network analysis of directed networks provided group-level information including weighted degree centrality and density. Exponential Random Graph Models fitted to these networks were used to consider the underpinning social processes including reciprocity, homophily, and the effect of individual attributes. Groups differed in the expression of behaviours, whilst, at the global level, density was significantly lower (*P* < 0.001) for the snout-snout (0.68 ± 0.15 SD) than snout-head (0.92 ± 0.04 SD) proximity networks. Statistically significant differences were also shown in the density across the contact networks (*P* < 0.001) with the lowest cohesion in the snout-snout (0.33 ± 0.14 SD), compared to the snout-head (0.52 ± 0.07 SD), and snout-body (0.66 ± 0.09 SD) contact networks. Familiarity was a predictor of interaction (*P* = 0.0001) across behaviours. Familiar pigs were nearly twice as likely to assort in the contact networks and three times more likely to assort in the snout-snout and snout-head proximity networks. Sex was not a predictor of snout proximity; however, females received significantly less behaviour than males in the snout-snout (odds ratio (**OR**): 0.78, *P* = 0.046), and snout-head (OR: 0.69, *P* = 0.001) contact networks. Snout proximity behaviours showed significant reciprocity (snout head: OR = 2.56; *P* = 0.008; snout-snout: OR = 2.80; *P* = 0.0001). Contact behaviours showed significant reciprocity in the snout-snout (OR = 2.40; *P* = 0.0001), and snout-head (OR = 1.55; *P* = 0.004) networks. Our study highlights behavioural nuances, with groups differing in snout proximity and contact patterns, in which reciprocation is normal behaviour, and snout-snout proximity and snout-snout contact are the least observed. Furthermore, it shows the influence of attributes on network structure to inform grouping strategies.

## Highlights

• Spatial proximity may be a poor indicator of social relationships in pigs.
• Social nosing presents as an alternative behaviour to indicate affiliation.
• Snout-head proximity shown as a non-discriminatory behaviour after mixing.
• Snout-snout contact shown as a selective behaviour driven by familiarity.
• Social nosing patterns can inform regrouping strategies to reduce aggression.

## Implications

Social relationships are important aspects of animal welfare but are often measured by spatial proximity, which may be a poor indicator for intensively housed animals. Instead, social nosing behaviour might be a better indicator of relationships in pigs. Using social networks, we showed that snout-head proximity is common and non-discriminatory, but that snout-to-snout contact is expressed selectively to familiar individuals. This information may aid in reducing aggression within the context of regrouping. Further, we show that using exponential random graph models can contribute to understanding animals’ social dynamics.

## Introduction

Understanding animal communication is necessary to determine the function and biological significance of behaviour (Forrester, 2008). While the expression of behaviour such as aggression is often overt and its cause and function well-researched, our interpretation of many other social behaviours is less clear (Rault, 2012). Subtle behaviours can easily become subject to interpretation, while their occurrence can have multiple underlying motivations. For example, close social contact may imply an affiliative relationship (Mendonça et al., 2021), the need for thermal comfort (Van Os et al., 2024), information gathering (Sawalhah et al. 2024) or all three. In animal welfare science, conclusions about the animals’ welfare heavily rely on their behavioural expression, and therefore, a correct interpretation of behaviours is essential. With the rising interest in positive animal welfare (Rault, 2012), the need to determine positive or affiliative social signals correctly is critical to the progress of this research field.

Social nosing has been suggested as a potential affiliative behaviour in pigs (O’Malley et al., 2022), and recent research has begun to decipher the nuances of nosing behaviour (Clouard et al., 2022, Clouard et al., 2024). The term social nosing has been described as a range of snout-related social behaviours including snout-to-snout contact and snout-body contact (Camerlink and Turner, 2013). However, it has also been emphasised that social nosing has multiple functions in pigs, including social recognition (Camerlink and Turner, 2013). Nosing between conspecifics occurs at consistent levels across time in stable social groups (Seddaiu et al., 2024), yet social context may impact these interactions. Studies show an increase in the amount of social nosing during the grouping of unfamiliar pigs (Kristensen et al., 2001, O’Malley et al., 2022), as they face a greater need to recognise individuals to establish a dominance hierarchy (Stookey and Gonyou, 1998), which may also increase agonistic encounters (O’Malley et al., 2022). Social nosing has a broad function in social behaviour and shows weak relationships with agonistic or damaging interactions (Camerlink and Turner, 2013, O’Malley et al., 2022).

At the same time, social nosing is also part of the affiliative behavioural repertoire, as it plays a key role in allo-grooming (Meynhardt, 1982, Seddaiu et al., 2024). Aggregating variations in social nosing and proximity, such as the distinction between snout-snout contact and snout-snout proximity, may result in contradictory outcomes and lead to misinterpretation (Camerlink et al., 2022). However, studies rarely distinguish between different forms of nosing behaviour, resulting in a lack of information on this aspect of social behaviour. As social nosing may be biologically relevant to the point where it influences the growth performance of pigs (Camerlink et al., 2012), it is meaningful to address this knowledge gap. Consideration must also be given to other attributes, which may potentially influence social nosing patterns, as individual differences may impact social processes and behavioural responses (e.g., Geverink et al., 2002, Munsterhjelm et al., 2019). For example, sex differences have been shown to influence social behaviour (e.g., Clouard et al., 2022), which is therefore important to account for when exploring social behaviours in mixed-sex groups.

Social behaviour is dynamic, and individuals will be influenced by their social partners. Social network analyses have been increasingly applied to quantify complex social structures, including in farm animals (e.g., De Freslon et al., 2019, Büttner et al., 2020, Gómez et al., 2022). Exponential random graph models (**ERGMs**) provide a statistical approach to model social networks by considering the underlying network processes that may be driving ties between individuals (Lusher et al., 2013a, Silk and Fisher, 2017). This can include, for example, measures of homophily (when individuals of a similar trait affiliate) (Borgatti et al., 2018a), reciprocity (when social ties are mutual), and transitivity (when a similar trait between two individuals is likely to develop a further tie with a third individual) (Borgatti et al., 2018b). To date, behavioural ecologists have successfully applied exponential random graph models to study how networks influence social and biological functions in a range of wild taxa. For example, a pioneering study used exponential random graph models to investigate the underlying attributes that predict dominance interactions (Dey and Quinn, 2014). Further examples show the effectiveness of exponential random graph models when considering patterns of social cohesion driven by affiliative behaviours (Wey and Blumstein, 2010, Kusch and Lane, 2021). However, the technique is currently underrepresented in the study of farmed animal social networks (De Freslon et al., 2019, Leung et al., 2023).

The objective of our study is to gain a deeper insight into the differences between various subtle social nosing behaviours, particularly the difference between snout proximity and snout contact. Earlier work suggested that snout proximity is used for social recognition (Gonyou, 2001), and thereby expressed regardless of social preferences, whereas snout contact may have a more affiliative function driven by existing social discrimination (Newberry and Wood-Gush, 1986). Our study further investigates the effects of homophily based on familiarity, reciprocity (mutual ties), and the effects of individual attributes (sex) on the likelihood of interactions occurring. We apply social network analysis and exponential random graph models to quantify the social structure and assess the importance of different social nosing behaviours. Social behaviour is studied in the days after mixing groups of finishing pigs, as this is expected to result in a high frequency of social nosing behaviours (Clouard et al., 2022, Clouard et al., 2023). We predict that snout proximity and snout contact networks will differ in their network metrics and will be driven by individual attributes and familiarity.

## Material and methods

### Animals and housing

The study was conducted from January 2023 to July 2023 at the SRUC pig unit (Easter Howgate, UK) in which the behavioural interactions of three batches of Large White × Landrace × Danish Duroc finishing pigs were observed, with approximately 50:50 male: female ratio per batch. In total 117, 12-week-old pigs (61 entire males and 56 females), housed across eight groups after regrouping, were observed. Prior to the study, from weaning (5 weeks of age), all pigs were moved from their original litter to be housed in mixed-litter groups, consisting of two sets of related pigs (approximately four pigs in each set). This allowed for familiarisation to occur with non-kin for 7 weeks. Following this familiarisation period, at 12 weeks old, pigs were then mixed again, into the groups observed in the current study. The groups of 3–4 siblings remained the same throughout the study whereas the overall group composition differed in each regrouping so that pigs were only familiar to their kin and unfamiliar to others. The average weight at the start of the study was 44.9 kg ± 5.54 (mean ± SD). Each group housed on average 15 pigs (range 14–16) and was composed of four groups of 3–4 siblings with an approximately equal male: female ratio. Pens measured 1.8 × 5.3 m (0.65 m^2^/pig) comprising a solid concrete floor partially covered with straw. Pens were cleaned daily with a refreshment of 4.5 kg straw between 0900 and 1000 h. Pigs had access to three nipple drinkers and one dry pellet feeder allowing for four individuals to eat simultaneously. Food was provided *ad libitum*. The feeders were replenished at 0900 h, and daily welfare checks were conducted between 1100 and 1300 h. The light was provided by a combination of natural light and strip lighting from 0700 until 1800 h. The walls of the pens were partially solid and partially gated, allowing pigs visual and snout contact with animals in adjacent pens. For individual identification, pigs were spray-marked with a blue symbol on their backs using a stock marker spray (East Riding Farm Services, Driffield, UK) and markings were refreshed daily.

### Behavioural observations

Daytime behaviour (0700–1800 h) was recorded onto a DVR (Hikvision 8 channel DVR, 7200 series) from two cameras per pen (Hikvision Turbo HD TV1 1080p resolution). Cameras were positioned to cover the whole pen including the resting area, drinking station, and feeder. Video observations were conducted on the day of regrouping (Day 1) and the following day (Day 2). These days were selected due to the re-establishment of social structure following a disruption event (Hemsworth et al., 2013). Focal animal sampling was applied to all animals in the groups. Observations consisted of four blocks of 15 min of continuous observation per focal pig per day resulting in 1 h per day and a total of 2 h per individual across the two observation days. Prior to the start of data collection, the videos were inspected for the time slots where pigs were active and thus engaging in social interactions. The optimal activity times (for all batches) selected for Day 1 were 1130, 1445, 1530, 1630 h and Day 2 were 0700, 0915, 1300, 1600 h. Observation times varied between the 2 days, a result of differences in activity levels between the day of mixing (all pigs were introduced to the new pen environment at 1100 h on Day 1) and the next consecutive day. On the day of mixing, pigs were more active in the hours directly following introduction to the new social group, a finding observed in previous research (Ott et al., 2014). Behaviour was observed using the ethogram in Table 1, thereby distinguishing whether the pig’s snout (the area from below the eyes, including the nose disc) made physical contact with another pig or whether the snout was in close proximity (< 30 cm) to another pig but not in contact. The distance for close contact was based on the length of the head (i.e., being less than a head length away). For all behaviours, the actor and recipient were noted. A single observer conducted observations.

**Table 1:** Ethogram for pig social nosing behaviours (Source: Camerlink and Turner, 2013, copyrights obtained).

Behaviour of focal pig	Definition
Snout-head proximity	Sniffing of the head, including the ears, and excluding the snout within ≤30 cm of another conspecific.
Snout-snout proximity	Sniffing the snout of another conspecific to within ≤30 cm without physical contact.
Snout-head contact	Snout contacts of another conspecific, excluding the snout and including any part of the head, including the ears.
Snout-snout contact	Snout contacts the snout of another conspecific.
Snout-body contact	Snout contacts with any part of another conspecific’s body, excluding the head, ears, and snout.

### Social network construction and analysis

We constructed directed and weighted social networks for each of our five behaviours (Table 1), with edges weighted based on the frequency of interactions. For every individual in each network, a count of received behaviours (weighted indegree centrality) and initiated behaviours (weighted outdegree centrality) were also recorded. For each network, we could also calculate its density (proportion of all dyadic interactions that occurred). Social network metrics of degree centrality were calculated in Ucinet 6, version 6.634 (Borgatti et al., 2002). To compare edge density between the behaviours, Linear Mixed Models were performed separately for the snout proximity and snout contact networks. Data were checked for normality using the Shapiro-Wilk test and histogram. Edge density was fitted as the response variable with behaviour type included as a categorical fixed effect and pen included as a random intercept. Linear Mixed Models were performed in R version 4.1.3 (R Development Core Team, 2020) using the ‘lme4′ package (Bates et al., 2015) and fitted with a posthoc Bonferroni correction to calculate the statistical significance of the fixed effects. The CV was calculated in Excel (Microsoft Corporation, 2018).

### Exponential random graph models

We used ERGMs to assess how well a combination of individual attributes (sex), previous social history (familiarity), and social processes (reciprocity) could explain the social network structure for each of our five behaviours. ERGMs are a family of statistical models designed specifically for analysing social network data that treat the network edges as random variables to be estimated (Lusher et al., 2013b). The variable defined as “Familiarity” was included to consider the effect of social mixing of individuals prior to remixing, interactions that could be considered as likely to increase the likelihood of any type of association with known conspecifics when entering a new social environment which includes unfamiliar pigs (Puppe, 1998, Kranz et al., 2022). Pig data were coded categorically using data related to social housing history, in which individuals were assigned a pen code related to housing prior to the study (Eth2_Pen: EP1 to EP7). The eight groups were mixed sex, with differences shown previously in social behaviour influenced by sex (Seyfang et al., 2018); we also included the categorical variable “Sex” in the models to include male (M) and female (F). Structural effects included the term “Mutual” to test the strength of the tendency for reciprocity in the different social nosing networks.

### Model construction and analysis

We tested the *P* of increased association based on homophily by previous social history using the “nodematch” function, with familiarity as the covariate. The “nodematch” function tests if an edge is more likely to exist between nodes that share an assigned common attribute. The *P* of engaging in a specified behaviour was tested using the “nodeofactor” function for initiated behaviour by males and the “nodeifactor” for received behaviour by females, with sex as the covariate. This approach is consistent with related research (de Freslon et al., 2019) and is appropriate when considering the differences between initiated male behaviour and received female behaviour in pigs (Seyfang et al., 2018). The “mutual” function was applied to establish the likelihood of symmetric ties occurring between individuals. To overcome the challenge of non-overlapping groups in a single model, structural zeros were used, allowing the model estimates to apply to all eight groups (constraints = ∼blockdiag(“Pen”)). Group 1 in each behavioural network represents the reference group for which all other group estimates are compared within the model. Parameter estimations in models consisting of all variables including mutuality were achieved with the application of the Monte Carlo Maximum Likelihood Effects. The goodness of fit and degeneracy were applied to the models using the ‘mcmc.diagnostic’ function (Geyer, 1992) to check that the models were well-fit (Supplementary Figures S1–S5). Exponential Random Graph Models were performed in R version 4.1.3 (R Development Core Team, 2020) using the ‘ergm’ package (Hunter et al., 2008, Krivitsky et al., 2023, Handcock et al., 2025).

## Results

### Snout proximity: network properties

**Fig. 1:**
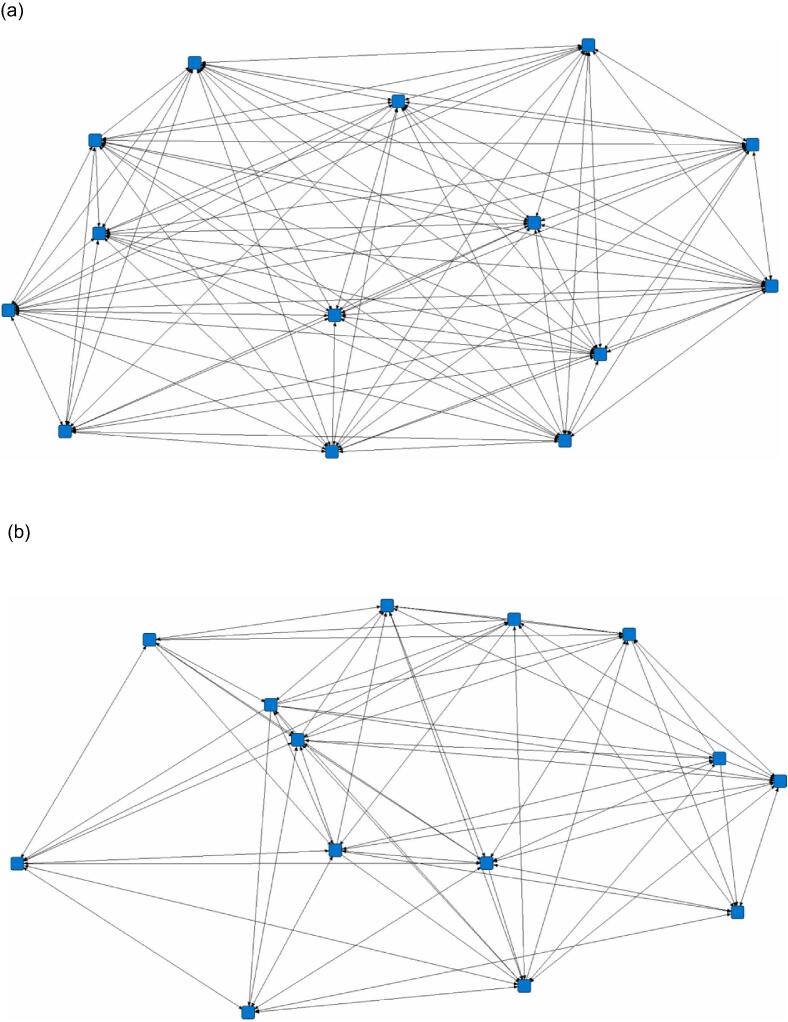
Sociograms illustrate differences in the density of pig interactions for (a) snout-head proximity (166 edges, 0.91 density) and (b) snout-snout proximity (92 edges, 0.51 density) networks for pen 7 (pigs = 14).

**Fig. 2:**
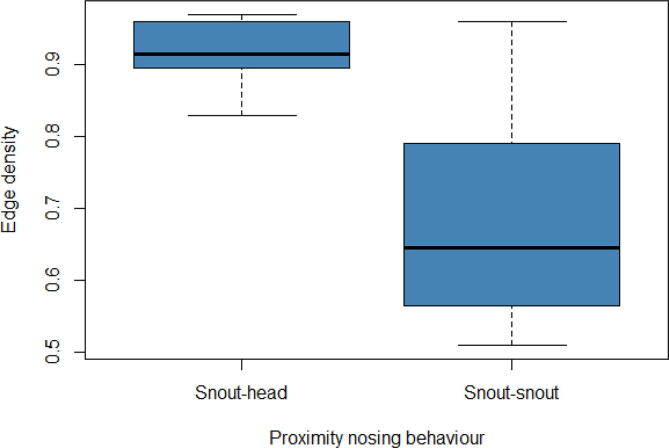
Distribution of the edge density for pig interactions across the snout-head proximity and snout-snout proximity networks. The snout-head proximity networks had a median edge density of 0.92, with a maximum edge density of 0.97 and a minimum edge density of 0.83, giving a range of 0.14. The snout-snout proximity networks had a median edge density of 0.65, with a maximum edge density of 0.96 and a minimum edge density of 0.51, giving a range of 0.45.

The number of edges in the snout-snout proximity networks was consistently lower than in the snout-head proximity networks (Table 2). A representative example (pen 7) of differences in density between the snout-head proximity (Fig. 1a) and snout-snout proximity (Fig. 1b) networks is illustrated. All groups in the snout-head proximity networks were cohesive and well-connected, as shown by the high edge density. A reduction in the chance of a tie between pigs in the snout-snout proximity compared to the snout-head proximity networks is shown by a statistically significant effect of snout-snout proximity behaviour on edge density (β = −0.23, SE = 0.04, *P* < 0.001). Bonferroni correction showed significantly lower density (*P* = 0.001) in the snout-snout proximity networks (0.68 ± 0.15 SD) compared with the snout-head proximity networks (0.92 ± 0.04 SD) (Fig. 2). At the group level, behavioural variation was also shown across groups with a higher CV in degree centrality in the snout-snout proximity networks (CV = 0.55) compared to the snout-head proximity networks (CV = 0.25).

**Table 2:** Proximity nosing behaviour of pigs at the group level (n = 8 groups of on average 15 pigs each). Social network metrics include the number of edges, density, and mean degree centrality (± SD). * Denotes statistically significant differences in density between snout-head proximity and snout-snout proximity (*P* < 0.001).

	Snout proximity					
	Snout-head			Snout-snout		
Item	Edges	Density	Degree	Edges	Density	Degree
Group						
1	175	0.96	143.6 ± 26.9	175	0.96	147.7 ± 30.1
2	166	0.91	123.6 ± 23.6	131	0.72	48.1 ± 12.9
3	185	0.88	103.1 ± 32.5	117	0.56	41.2 ± 14.3
4	151	0.83	87.9 ± 37.7	104	0.57	40.0 ± 19.2
5	204	0.97	148.0 ± 28.4	172	0.82	74.9 ± 21.2
6	230	0.96	207.1 ± 44.5	182	0.76	81.0 ± 25.4
7	166	0.91	120.6 ± 26.2	92	0.51	31.7 ± 11.2
8	193	0.92	152.3 ± 45.2	119	0.57	52.7 ± 24.2
Mean	183.8 ± 23.5	0.92 ± 0.04*	136.1 ± 47.8	136.5 ± 32.7	0.68 ± 0.1*	64.7 ± 40.5

### Snout proximity: exponential random graph models

Model estimates for the snout-head proximity and snout-snout proximity networks are presented in Table 3. Familiarity but not sex predicted unweighted edges for both snout proximity behaviours. Homophily by familiarity was positive and highly statistically significant in both snout proximity models. Demonstrating that pigs that had been previously housed together were around three times more likely to engage in snout-head proximity interactions and 5% more likely to engage in snout-snout proximity interactions within the first 48 h of regrouping than unfamiliar pigs. Sex did not influence the likelihood of engaging in an interaction in either the snout-head proximity or snout-snout proximity networks indicating that neither males nor females were more likely to initiate these behaviours. Social interactions also showed statistically significant reciprocity in both networks, such that interactions were nearly three times more likely to occur when both pigs in a dyad initiated these behaviours towards each other (Table 3). In both the snout proximity networks, there was an approximately greater than 50% chance of a potential directed interaction to occur between two individuals. The snout-head proximity networks were shown to be considerably denser across all groups than the snout-snout proximity networks. In the snout-snout proximity networks, the edges in four of the groups were found to be statistically significantly less likely to occur compared to the reference group (Group 1).

**Table 3:** Model estimates (± SE), odds ratio (OR), and statistical significance for the exponential random graph models predicting the formation of a within-group proximity interaction in the snout-head and snout-snout pig networks. Group 1 represents the reference group (n = 8 groups of on average 15 pigs each). * Denotes statistically significant differences in behaviour between pens compared to the reference group (Group 1), homophily between familiar and unfamiliar pigs, within sex attributes, and where reciprocity of behaviour is significant.

	Snout proximity					
	Snout-head			Snout-snout		
Item	Estimate (SE)	OR	*P-*value	Estimate (SE)	OR	*P-*value
Group						
1	2.32 (0.5)	10.18	0.0001*	0.63 (0.2)	1.88	0.004*
2	−0.37 (0.2)	−	0.106	−0.19 (0.1)	−	0.095
3	−0.55 (0.2)	0.58	0.009*	−0.48 (0.1)	0.62	0.0001*
4	−0.07 (0.2)	0.93	0.001*	−0.46 (0.1)	0.67	0.0001*
5	0.16 (0.3)	−	0.565	0.05 (0.1)	−	0.663
6	0.02 (0.2)	−	0.921	−0.10 (0.1)	−	0.363
7	−0.37 (0.2)	−	0.011	−0.48 (0.1)	0.67	0.0001*
8	−0.35 (0.2)	−	0.126	−0.46 (0.1)	0.67	0.0001*

Homophily						
Familiarity	1.16 (0.4)	3.19	0.001*	0.56 (0.2)	0.57	0.0002*

Attributes						
Male	−0.27 (0.2)	−	0.170	−0.10 (0.1)	−	0.411
Female	−0.007 (0.2)	−	0.971	−0.10 (0.1)	−	0.391

Reciprocity						
Mutual	0.94 (0.4)	2.56	0.008*	1.03 (0.2)	2.80	0.0001*

### Snout contact: network properties

**Fig. 3:**
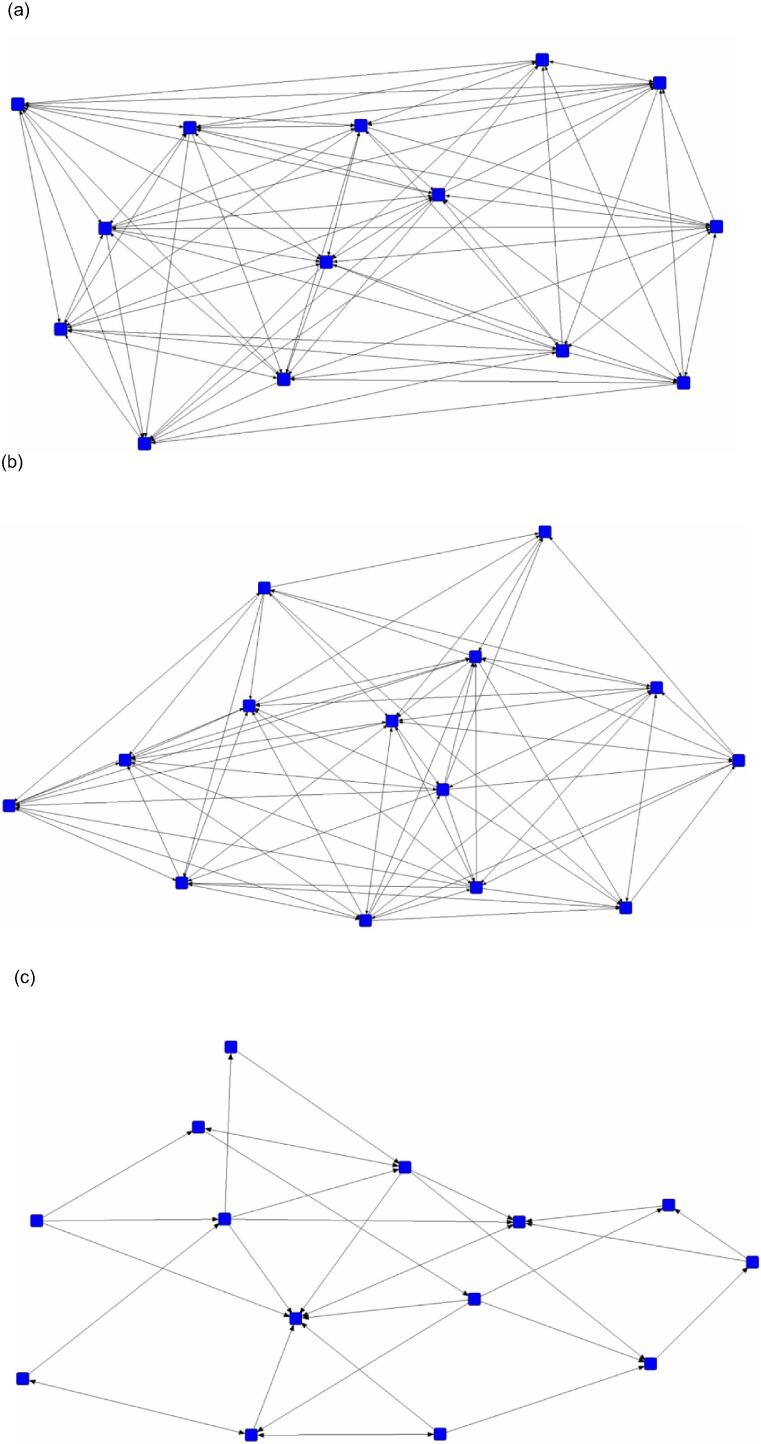
Sociograms illustrate differences in the density of pig interactions for the (a) snout-body contact (103 edges, 0.57 density), (b) snout-head contact (84 edges, 0.46 density), and (c) snout-snout contact (31 edges, 0.17 density) networks for pen 7 (pigs = 14).

**Fig. 4:**
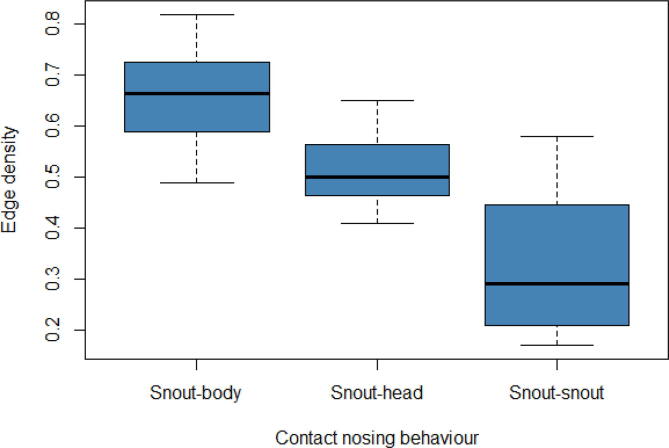
Distribution of the edge density for pig interactions across the snout-body contact, snout-head contact, and snout-snout contact networks. The snout-body contact network had a median edge density of 0.67, with a maximum edge density of 0.82 and a minimum edge density of 0.49, giving a range of 0.33. The snout-head contact networks had a median edge density of 0.50, with a maximum edge density of 0.65 and a minimum edge density of 0.41, giving a range of 0.24. The snout-snout contact networks had a median edge density of 0.29, with a maximum edge density of 0.58 and a minimum edge density of 0.17, giving a range of 0.41.

The snout-snout contact networks (Table 4) were found to be the least well-connected of the contact behaviours. A representative example (pen 7) of differences in density between the snout-body contact (Fig. 3a), snout-head contact (Fig. 3b), and snout-snout contact (Fig. 3c) networks is illustrated. This is further shown in the statistically significant effect of specific nosing interactions on edge density found across the contact behaviours (β = 0.52, SE = 0.04, *P* < 0.001). Bonferroni correction showed the snout-body contact networks (0.66 ± 0.09 SD) had significantly higher density (*P* < 0.001) than the snout-snout contact networks (0.33 ± 0.14 SD). The snout-snout contact networks were also found to be significantly less cohesive (*P* = 0.01) than the snout-head contact networks (0.52 ± 0.07 SD) (Fig. 4). At the group level, behavioural variation was again shown across groups with a higher CV in degree centrality in the snout-snout contact (CV = 0.47), compared to the snout-head contact (CV = 0.22) and snout-body contact (CV = 0.19) networks.

**Table 4:** Contact nosing behaviour of pigs at the group level (n = 8 groups of on average 15 pigs each). Social network metrics include the number of edges, density, and mean degree centrality (± SD). * Denotes statistically significant differences in density between snout-body contact and snout-snout contact (*P* < 0.001), and between snout-snout contact, and snout-head contact (*P* = 0.01).

	Snout contact								
	Snout-body			Snout-head			Snout-snout		
Item	Edges	Density	Degree	Edges	Density	Degree	Edges	Density	Degree
Group									
1	149	0.82	64.1 ± 11.5	106	0.58	30.1 ± 9.0	105	0.58	28.1 ± 5.9
2	135	0.74	59.0 ± 10.4	119	0.65	46.1 ± 12.0	94	0.52	26.0 ± 8.2
3	129	0.61	48.0 ± 14.1	98	0.47	27.2 ± 7.2	42	0.20	9.5 ± 3.2
4	89	0.49	29.3 ± 12.6	75	0.41	25.9 ± 13.4	40	0.22	9.3 ± 5.5
5	150	0.71	55.1 ± 15.1	106	0.51	32.8 ± 8.7	74	0.35	16.3 ± 4.7
6	158	0.66	56.5 ± 15.3	133	0.55	47.1 ± 11.3	89	0.37	18.9 ± 7.4
7	103	0.57	46.3 ± 15.2	84	0.46	30.9 ± 9.0	31	0.17	7.1 ± 3.0
8	141	0.67	55.3 ± 17.3	102	0.49	37.9 ± 20.3	48	0.23	11.7 ± 7.8
Mean	131.8 ± 22.6	0.66 ± 0.1*	51.8 ± 17.3	102.9 ± 17.1	0.52 ± 0.1*	34.7 ± 14.2	65.4 ± 26.7	0.33 ± 0.1*	15.7 ± 9.5

### Snout contact: exponential random graph models

Model estimates for the snout-body contact, snout-head contact, and snout-snout contact networks are presented in Table 5. Homophily by familiarity influenced all three contact networks, further indicating that familiar pigs were around twice as likely to engage in contact interactions compared with unfamiliar pigs. Sex was only found to be a statistically significant predictor of interactions for females and not male pigs in the snout-head contact and snout-snout contact networks. Female pigs were 69% less likely to receive snout-head contacts and 78% less likely to receive snout-snout contacts than male pigs. Social interactions also showed positive and statistically significant reciprocity of behaviour in the snout-snout contact and snout-head contact networks, with interactions around twice as likely to occur when both pigs in a dyad initiated these behaviours towards each other. In the snout-snout contact reference group (Group 1), there was an approximately 50% chance of a potential directed interaction to occur between two individuals. However, in six out of seven of the other groups, edges were statistically significantly less likely to occur, corresponding to these networks being less densely connected (Table 4). A similar pattern of behaviour is observed in the snout-head contact networks. In contrast, snout-body contact across groups indicated a greater than 50% chance of a potential directed interaction except for Group 4.

**Table 5:** Model estimates (± SE), odds ratio (OR), and statistical significance for the exponential random graph models predicting the formation of a within-group contact interaction in the snout-body, snout-head, and snout-snout pig networks. Group 1 represents the reference group (n = 8 groups of on average 15 pigs each). * Denotes statistically significant differences in behaviour between pens compared to the reference group (Group 1), homophily between familiar and unfamiliar pigs, within sex attributes, and where reciprocity of behaviour is significant.

	Snout contact								
	Snout-body			Snout-head			Snout-snout		
Item	Estimate (SE)	OR	*P-*value	Estimate (SE)	OR	*P-*value	Estimate (SE)	OR	*P-*value
Group									
1	1.66 (0.3)	5.24	0.0001*	0.15 (0.2)	−	0.416	−0.16 (0.2)	−	0.395
2	−0.20 (0.1)	−	0.141	0.16 (0.1)	−	0.146	−0.08 (0.1)	−	0.428
3	−0.52 (0.1)	0.59	0.0001*	−0.20 (0.1)	0.82	0.035*	−0.70 (0.1)	0.49	0.0001*
4	−0.94 (0.1)	0.39	0.0001*	−0.34 (0.1)	0.72	0.001*	−0.56 (0.1)	0.57	0.0001*
5	−0.29 (0.1)	0.75	0.024*	−0.14 (0.1)	−	0.154	−0.40 (0.1)	0.67	0.0001*
6	−0.41 (0.1)	0.67	0.001*	−0.07 (0.1)	−	0.463	−0.29 (0.1)	0.75	0.005*
7	−0.61 (0.1)	0.54	0.0001*	0.20 (0.1)	0.82	0.047*	−0.77 (0.1)	0.46	0.0001*
8	−0.38 (0.1)	0.69	0.021*	−0.17 (0.1)	−	0.076*	−0.57 (0.1)	0.57	0.0001*

Homophily									
Familiarity	0.80 (0.2)	2.24	0.0001*	0.81 (0.1)	2.25	0.0001*	0.72 (0.1)	2.06	0.0001*

Attributes									
Male	−0.13 (0.1)	−	0.250	−0.04 (0.1)	−	0.721	−0.09 (0.1)	−	0.438
Female	−0.19 (0.1)	−	0.094	−0.38 (0.1)	0.69	0.001*	−0.24 (0.1)	0.78	0.046*

Reciprocity									
Mutual	−0.20 (0.2)	−	0.227	0.44 (0.2)	1.55	0.004*	0.88 (0.2)	2.40	0.0001*

## Discussion

We investigated specific snout proximity and snout contact interactions in pigs to further inform our understanding of the behaviours that could be expected to represent affiliative ties and the attributes that promote behavioural interactions. We found marked differences in the network structures between the behaviours and detected the effects of familiarity and sex on interaction patterns after social instability. Familiarity was a strong predictor of interactions, with pigs that had previously been housed together before regrouping much more likely to interact in all the snout proximity and snout contact networks. Sex did not influence levels of received and initiated behaviours in the snout proximity networks, but females had a smaller chance of receiving snout-snout contact and snout-head contact compared with males. Reciprocal interactions within dyads were a key feature in all networks where pigs initiated snout proximity and snout contact to the snout or head of the recipient, but not the network related to body contact.

The social nosing proximity networks represented the most cohesive, well-connected social structures. This was particularly evident in the snout-head proximity networks in which all pens demonstrated high density. This high density is unsurprising given the need for pigs to re-establish a dominance hierarchy after the regrouping event. For this, social recognition is paramount, and pigs will need to be in close snout proximity (but not necessarily contact) to recognise each other through olfaction (Kristensen et al., 2001). Further to this, the dense networks for snout proximity are to be expected considering the space restrictions of the environment, where, on average, there was 0.65 m^2^ per pig. A recent study by Orsini et al. (2025) highlights the impact of environmental context on affiliation when considering the expected outputs of different social network metrics, particularly degree centrality which was found to be less affected by proximity measures compared to more complex measures of relationships and transitivity. Still, to date, proximity represents the principal measure of affiliation, both in pigs (Goumon et al., 2020, Jowett et al., 2024) and other farm species (Ozella et al., 2020, Gómez et al., 2022, Marina et al., 2024). However, proximity measures require care in interpretation under reduced spatial conditions, and when there is shared access to resources. Under these conditions, proximity may reflect social tolerance (Abeysinghe et al., 2013), or location preference rather than conspecific preference (Turner et al., 2003).

When considering the potential applications of the study, the findings highlight the significance of space allowance postmixing, a time when it is of increased importance to allow individuals the opportunity to engage in interactions with discrimination. Particularly, due to the meaningful difference between proximity and contact patterns, allowing pigs to distinguish between these interactions at mixing to reduce aggressive encounters and improve welfare. Further showing, that when there is limited space, snout-head proximity after mixing is not an effective representation of an affiliative relationship. The odds of engaging in snout-snout proximity were lower than the odds of engaging in snout-head proximity, potentially indicating selective decision-making. Snout-head proximity could be considered a generalised interaction while, in contrast, snout-snout proximity is more localised, underpinning communication, identification, and other complex processes that develop in early life (Ambruosi et al., 2024, Backeman Hannius et al., 2024). Snout-snout proximity behaviours showed a wider distribution of edges between groups than snout-head proximity behaviours and may indicate the variation of the densities shown at the group level across the behavioural networks.

Snout-snout contacts represented the behavioural interaction that occurred the least, with the network measures (degree centrality and density) and models supporting the finding that these networks were the least well-connected networks of the contact behaviours. Snout-snout interaction patterns may represent a trade-off during regrouping situations, as pigs adjust their snout proximity and contact behaviours depending on social stability (Camerlink et al., 2021, O’Malley et al., 2022). Our findings highlight marked differences in the network structure of snout proximity and snout contact behaviour, emphasising the importance of distinguishing nuances in social behaviour. Slight variations in interaction patterns across groups were also shown when considering the snout-head contact and snout-body contact networks. Further indicating the need to disentangle the nuances of interaction between domestic pigs, particularly when social disruption and instability are experienced, as the social context can significantly impact biological and behavioural responses (D’Eath, 2004, Coutellier et al., 2007).

Consistent with recent research, there was a lower expression of snout-snout contacts at the network level across the eight groups compared to the other contact behaviours. Clouard et al. (2024) found the expression of snout-snout contacts to be random in two mixed-litter groups in both the postweaning and finishing stage of development, with a lower mean of snout-snout contact per pig than snout-body contact, despite a relatively high density observed across the groups in both phases. This indicates that although there were opportunities for snout-snout interactions, these behaviours were not engaged at the same level as snout-body contacts. Furthermore, when considering opportunities for contact determined by surface body area, snout-body contacts would be explicitly more available for non-random interactions than snout-snout contacts. Particularly as snout-body interactions can occur fleetingly as a result of close proximity and potentially without the recipient’s knowledge of the interaction (for example, if the recipient is simply walking past the initiating, resting pig). As such, the networks in the current study show that compared with the other behaviours, snout-snout contact is a more selective behaviour. This is shown in a study by Camerlink and Turner (2013), in which nosing the body relates to various other social behaviours, including nosing of other body parts of the recipient. On the other hand, snout-snout contact (which in the previous study was referred to as nose-nose contact and included proximity) did not relate to other social behaviours. The selectiveness of this potentially affiliative behaviour (Camerlink and Turner, 2013), supported by the increased odds of a reciprocal interaction with a familiar conspecific, is in line with the selectiveness of affiliative relationships in pigs (Goumon et al., 2020). This further confirms the expectation that the frequency of snout-snout contact would be lower than other social nosing behaviours directly following mixing in the period when the social structure is being reestablished (Meese and Ewbank, 1973).

Understanding the role of familiarity and sex effects may provide enhanced insight into the significance of social nosing behaviours for pigs’ social relationships. Familiarity was a significant predictor of tie formation in all the snout proximity and snout contact networks. Familiar pigs were twice as likely to engage in any of the contact behaviours and three times as likely to have snout-head proximity. Furthermore, the lack of reciprocity in the snout-body contact networks supports the findings of Goumon et al. (2020) who reported snout-body interactions as asymmetrical. In related research, kinship is not shown to predict assortment by snout-snout or snout-body contacts. Clouard et al. (2024) showed no assortment by kinship when groups had a 2-week adaption phase, in which they would have become familiar, with new social networks prior to behavioural observations. This differs from the current study in which groups were comprised of individuals who were either familiar or not at all familiar. The findings potentially suggest that familiarity may be as important or equally as effective as kinship for the development of affiliative ties, with familiarity representing a good proxy of kinship during early life. The difference in social stability may also have influenced the frequency and type of social nosing behaviour shown, whereby snout-snout contacts after social disruption decreased in the weeks afterwards (O’Malley et al., 2022). Although our study only considered the first 2 days after mixing, the findings show familiarity increased the odds of snout-snout contact between individuals. In line with the literature discussed, it may be that familiarity is only important in the initial stages following social disruption. O’Malley et al. (2022) showed that all combined incidences of nose contact behaviours (nose-body, nose-head, nose-nose) reduced consistently in the observation periods which included the 3, 6, and 9-week time points following mixing. It is likely that under stable conditions, the function of snout proximity may change from information gathering to social maintenance and cohesion (Clouard et al., 2022). Furthermore, as spatial proximity is generally associated with strong social bonds (Massen et al., 2010), this may indicate that in the current study, nosing proximity between familiar pigs may have been influenced by locality and the immediacy of dynamic disruption.

Sex was not found to influence behavioural interactions in the snout proximity networks. However, in the snout-snout contact and snout-head contact networks, females were less likely to receive contact compared to males. This contrasts with the finding of Clouard et al. (2024), in which, overall, snout-snout and snout-body contact interactions were not influenced by sex. However, the study did indicate a trend towards a sex effect in specific pens. One potential explanation for the overall differences in the findings may lie in the selected observation phases, our study only considered behaviours in the finishing phase, the phase in which Clouard et al. (2024) proposed potential sex effects compared to the postweaning phase. As behaviours in the postweaning phase were not observed in our study, it is not possible to make direct comparisons or assumptions between the findings of both studies.

## Conclusion

This study shows that there are differences in the nuanced expression of social nosing behaviours in pigs. Although there was variation among groups, higher cohesion was found in the snout proximity than in the snout contact social networks. Snout-snout contacts were the least expressed behaviour. Homophily by familiarity was found to be an attribute that predicted ties under conditions of social instability, while there were limited effects of sex. Sniffing of the head and snout was reciprocated, which was not the case for sniffing of the body. The results show the importance of distinguishing subtle differences in nosing behaviour to better understand their potential function and importance to communication. It further provides support for the implementation of stricter definitions of affiliative behaviours, as determined by social nosing, when considering random and socially discriminatory behaviours under commercial housing conditions. Even under restricted space allowance, pigs maintain the distinction between these subtle behaviours, in accordance with the social context.

## Supplementary material

Supplementary Material for this article (https://doi.org/10.1016/j.animal.2025.101585) can be found at the foot of the online page, in the Appendix section.

## Ethics approval

The experiment was approved by the SRUC Animal Experiments Committee (RP 06-2022/AE 16-2022), and all regulated procedures were conducted under licence from the UK Home Office (Project Licence number: PP1403242) in line with the Animals (Scientific Procedures) Act 1986 (ASPA).

## Data and model availability statement

Data available at Turner, S.P., (2024). Dataset on pig social nosing − Scotland’s Rural College (SRUC): https://figshare.com/s/3080f0ef17ea39434699. Information can be made available from the authors upon request.

## Declaration of Generative AI and AI-assisted technologies in the writing process

During the preparation of this work the author(s) did not use any AI and AI-assisted technologies.

## Author ORCIDs

**Sarah Jowett:**https://orcid.org/0000-0001-9351-3793.

**Matthew Silk:**https://orcid.org/0000-0002-8318-5383.

**Victoria Lee:**https://orcid.org/0000-0003-3981-387X.

**Simon Turner:**https://orcid.org/0000-0001-9198-9448.

**Irene Camerlink:**https://orcid.org/0000-0002-3427-2210.

## CRediT authorship contribution statement

**S.L. Jowett:** Writing – review & editing, Writing – original draft, Visualisation, Methodology, Formal analysis, Data curation, Conceptualisation. **M.J. Silk:** Writing – review & editing, Methodology, Formal analysis. **V. Lee:** Writing – review & editing, Investigation, Conceptualisation. **S.P. Turner:** Writing – review & editing, Project administration, Funding acquisition. **I. Camerlink:** Writing – review & editing, Project administration, Funding acquisition.

## Declaration of interest

None.
