# Supplementary material for: Characterising nosing behaviours in pigs after mixing using social network analysis
Source: Animal. 2025 Aug;19(8):101585. doi: 10.1016/j.animal.2025.101585 (PMC12351059; doi:10.1016/j.animal.2025.101585)
Supplement: Supplementary Data 1 [file mmc1.pdf]

Journal : Animal

Characterising nosing behaviours in pigs after mixing using social network analysis

S.L. Jowett [a*], M.J. Silk [b], V. Lee [c], S.P. Turner [c], I. Camerlink [a].

[a] *Institute of Genetics and Animal Biotechnology, Polish Academy of Sciences, Postępu 36a, 05-552 Jastrzębiec, Poland.*

[b] *Institute of Ecology and Evolution, School of Biological Sciences, University of Edinburgh, Edinburgh, UK.*

[c] *Animal Behaviour & Welfare, Animal and Veterinary Sciences Department, Scotland's Rural College (SRUC), West Mains Rd., Edinburgh, EH9 3jG, UK.*

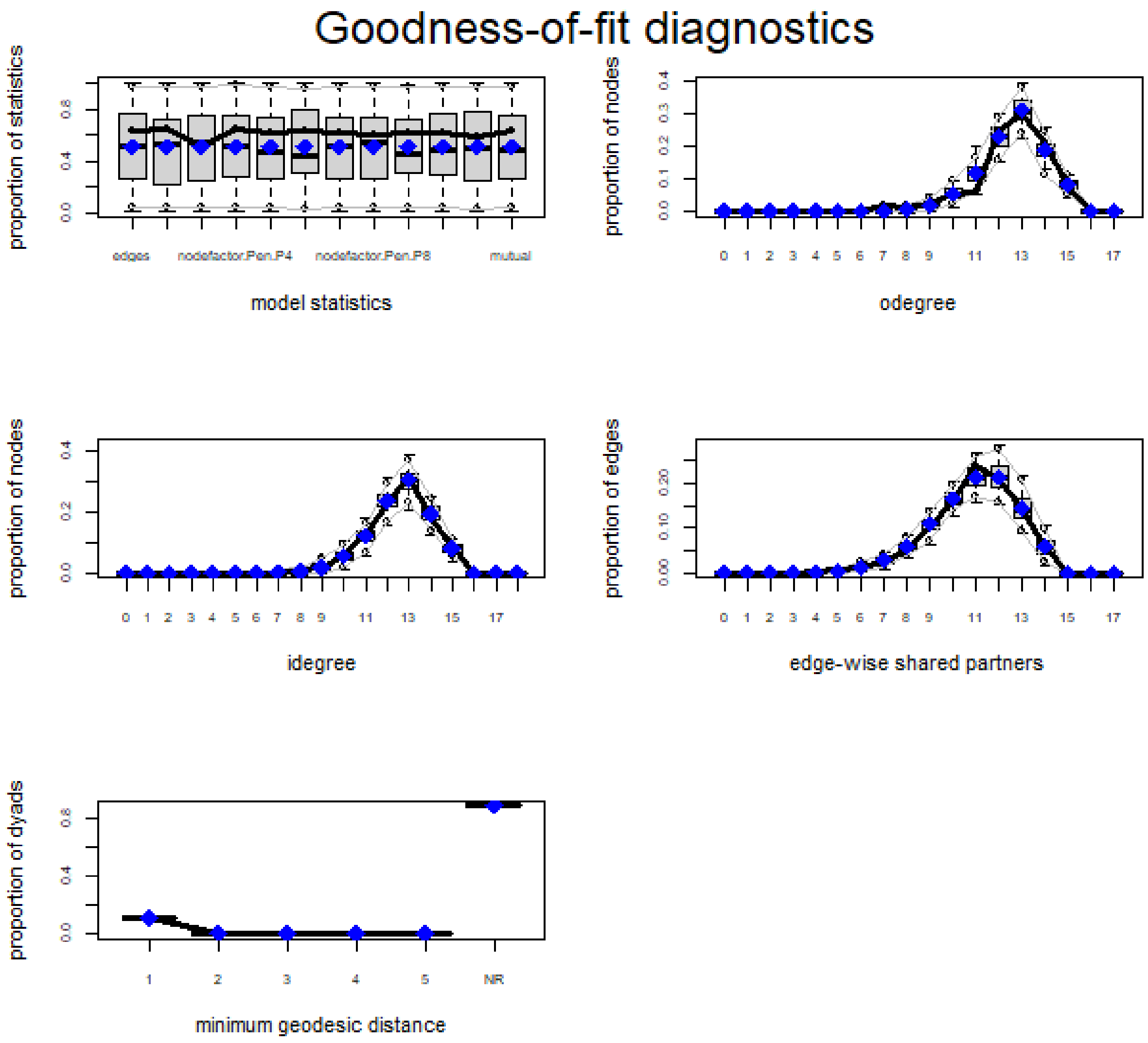

Supplementary Figure S1: Goodness-of-fit diagnostics for the snout-head proximity pig networks, odegree refers to outdegree centrality and idegree refers to indegree centrality.

Journal : Animal

Characterising nosing behaviours in pigs after mixing using social network analysis

S.L. Jowett [a*], M.J. Silk [b], V. Lee [c], S.P. Turner [c], I. Camerlink [a].

[a] *Institute of Genetics and Animal Biotechnology, Polish Academy of Sciences, Postępu 36a, 05-552 Jastrzębiec, Poland.*

[b] *Institute of Ecology and Evolution, School of Biological Sciences, University of Edinburgh, Edinburgh, UK.*

[c] *Animal Behaviour & Welfare, Animal and Veterinary Sciences Department, Scotland's Rural College (SRUC), West Mains Rd., Edinburgh, EH9 3jG, UK.*

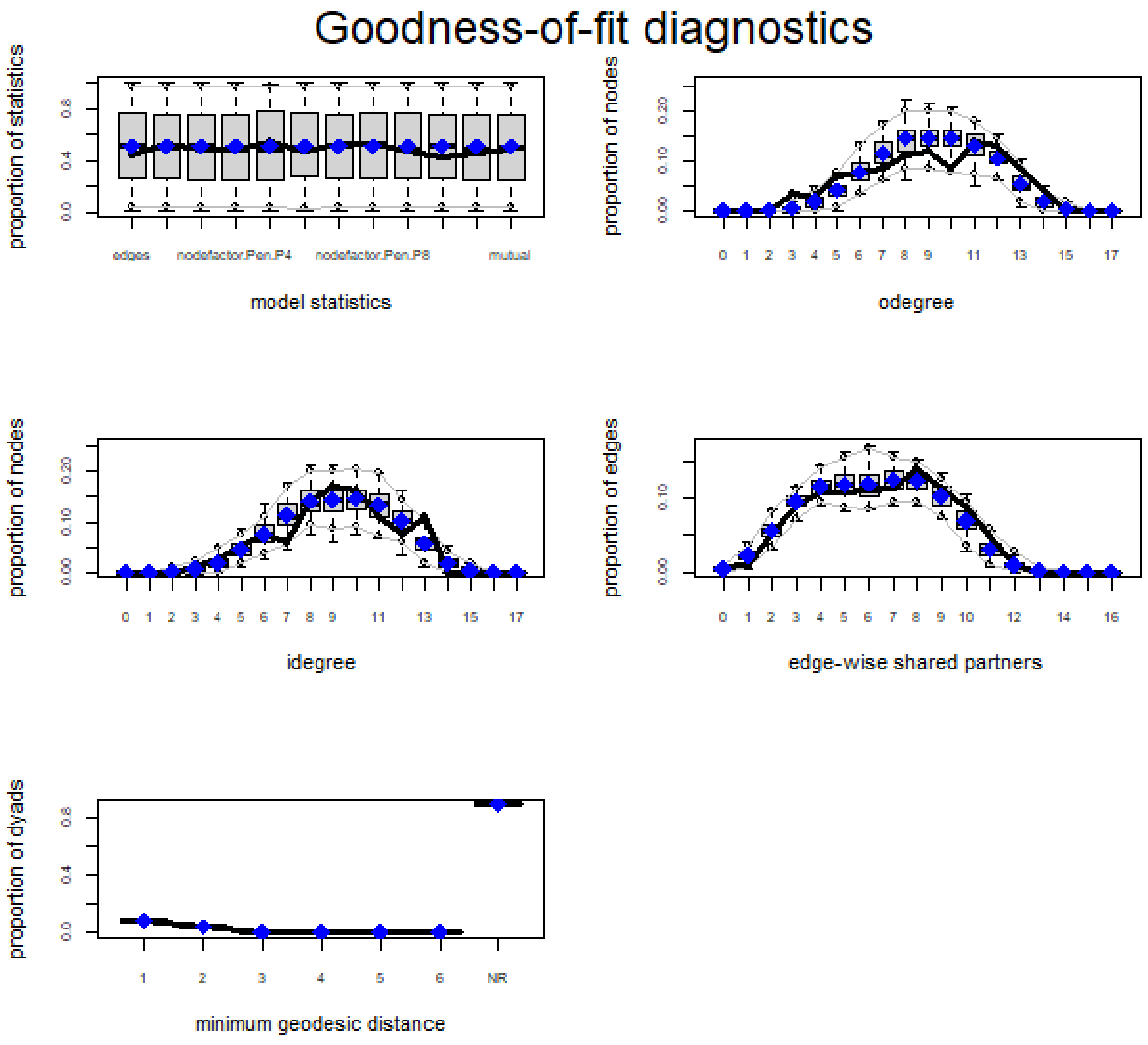

Supplementary Figure S2: Goodness-of-fit diagnostics for the snout-snout proximity pig networks, odegree refers to outdegree centrality and idegree refers to indegree centrality.

Journal : Animal

Characterising nosing behaviours in pigs after mixing using social network analysis

S.L. Jowett [a*], M.J. Silk [b], V. Lee [c], S.P. Turner [c], I. Camerlink [a].

[a] *Institute of Genetics and Animal Biotechnology, Polish Academy of Sciences, Postępu 36a, 05-552 Jastrzębiec, Poland.*

[b] *Institute of Ecology and Evolution, School of Biological Sciences, University of Edinburgh, Edinburgh, UK.*

[c] *Animal Behaviour & Welfare, Animal and Veterinary Sciences Department, Scotland's Rural College (SRUC), West Mains Rd., Edinburgh, EH9 3jG, UK.*

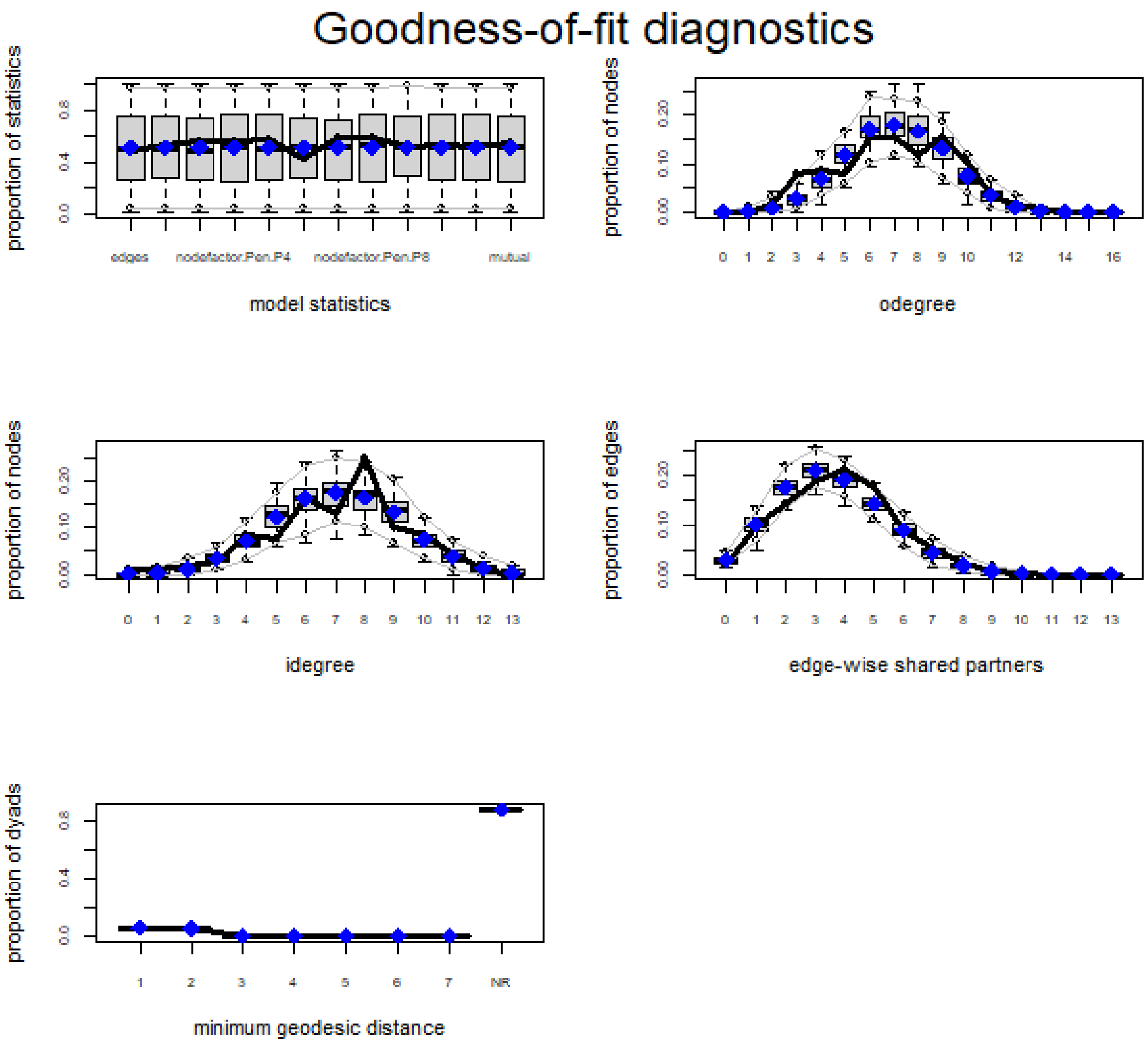

Supplementary Figure S3: Goodness-of-fit diagnostics for the snout-head contact pig networks, odegree refers to outdegree centrality and idegree refers to indegree centrality.

Journal : Animal

Characterising nosing behaviours in pigs after mixing using social network analysis

S.L. Jowett [a*], M.J. Silk [b], V. Lee [c], S.P. Turner [c], I. Camerlink [a].

[a] *Institute of Genetics and Animal Biotechnology, Polish Academy of Sciences, Postępu 36a, 05-552 Jastrzębiec, Poland.*

[b] *Institute of Ecology and Evolution, School of Biological Sciences, University of Edinburgh, Edinburgh, UK.*

[c] *Animal Behaviour & Welfare, Animal and Veterinary Sciences Department, Scotland's Rural College (SRUC), West Mains Rd., Edinburgh, EH9 3jG, UK.*

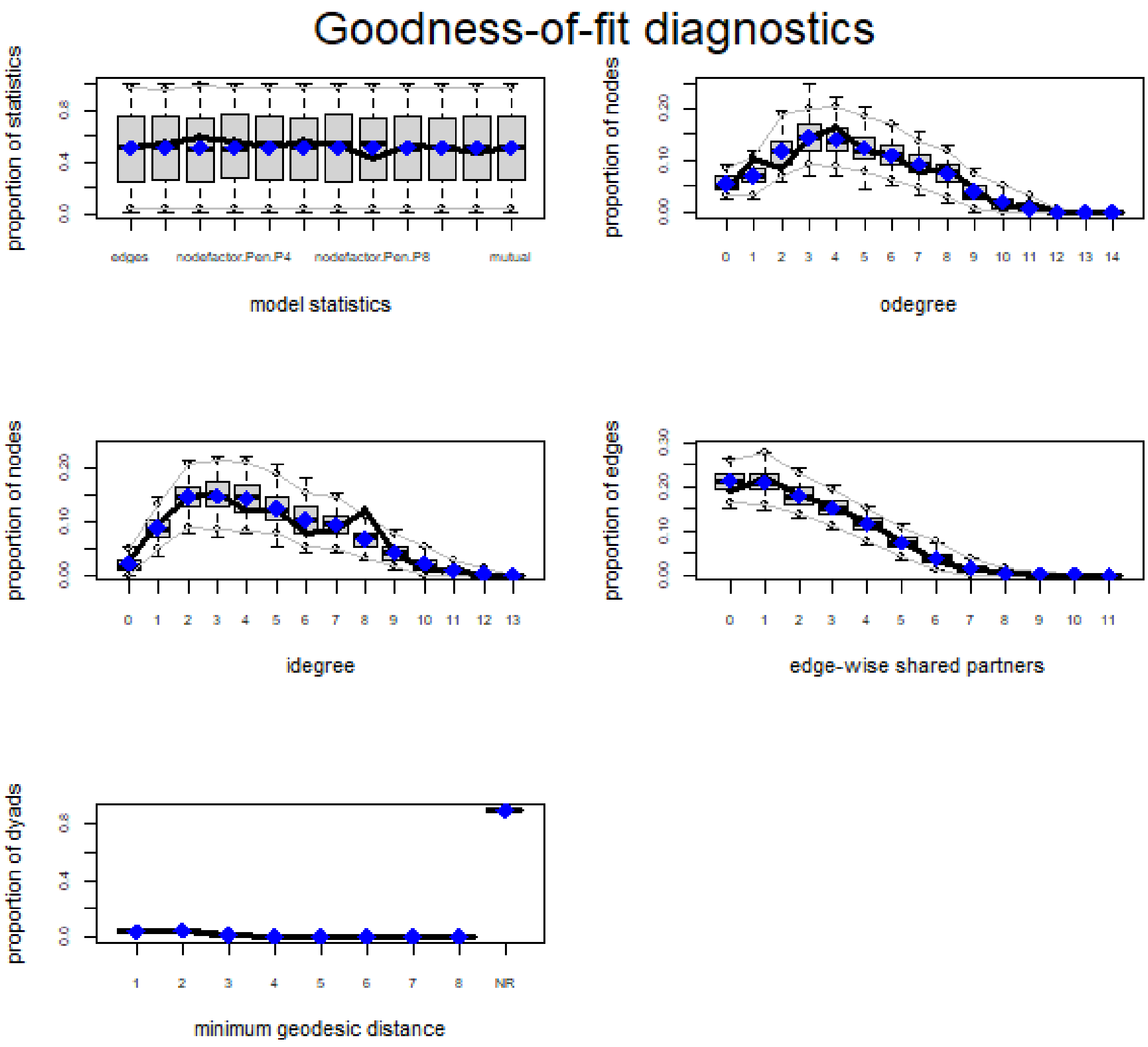

Supplementary Figure S4: Goodness-of-fit diagnostics for the snout-snout contact pig networks, odegree refers to outdegree centrality and idegree refers to indegree centrality.

Journal : Animal

Characterising nosing behaviours in pigs after mixing using social network analysis

S.L. Jowett [a*], M.J. Silk [b], V. Lee [c], S.P. Turner [c], I. Camerlink [a].

[a] *Institute of Genetics and Animal Biotechnology, Polish Academy of Sciences, Postępu 36a, 05-552 Jastrzębiec, Poland.*

[b] *Institute of Ecology and Evolution, School of Biological Sciences, University of Edinburgh, Edinburgh, UK.*

[c] *Animal Behaviour & Welfare, Animal and Veterinary Sciences Department, Scotland's Rural College (SRUC), West Mains Rd., Edinburgh, EH9 3jG, UK.*

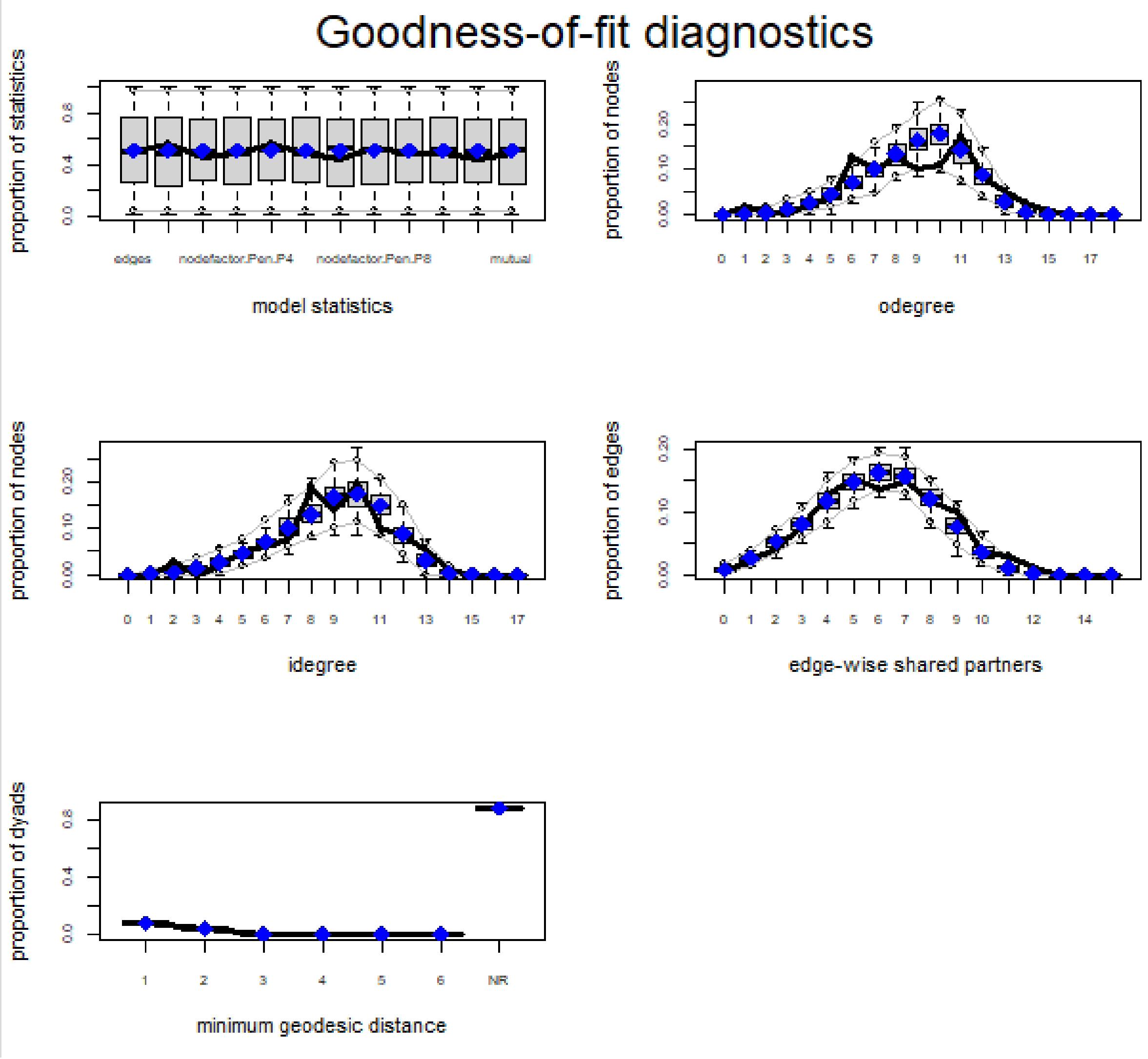

Supplementary Figure S5: Goodness-of-fit diagnostics for the snout-body contact pig networks, odegree refers to outdegree centrality and idegree refers to indegree centrality.
